# Long‐term outcome prediction for chronic thromboembolic pulmonary hypertension after pulmonary endarterectomy

**DOI:** 10.1002/clc.23900

**Published:** 2022-09-07

**Authors:** Wu Song, Jiade Zhu, ZhaoJi Zhong, Yunhu Song, Sheng Liu

**Affiliations:** ^1^ Department of Cardiac Surgery, Key Laboratory of Pulmonary Vascular Medicine, Fuwai Hospital, National Center for Cardiovascular Diseases Chinese Academy of Medical Sciences & Peking Union Medical College Beijing China; ^2^ Department of Cardiac Surgery, Guangdong Cardiovascular Institute, Guangdong Provincial People's Hospital Guangdong Academy of Medical Sciences Guangzhou China

**Keywords:** endarterectomy, long‐term survival, pulmonary hypertension, surgical, thromboembolism

## Abstract

**Background:**

The definitive treatment for chronic thromboembolic pulmonary hypertension (CTEPH) is pulmonary endarterectomy (PEA), which has good long‐term outcomes. However, after surgery, a quarter of the patients still have residual pulmonary hypertension (RPH). In pulmonary hemodynamics, there are no unified criteria for RPH, even though the level may affect long‐term survival.

**Methods:**

Between March 1997 and December 2021, 253 CTEPH patients were treated at our center with PEA. Patients were evaluated retrospectively and classified into early (1997–2014) and late (2015–2021) groups. The clinical characteristics and perioperative outcomes of the two groups were compared, and risk factor analysis for RPH and long‐term survival for all cases was performed.

**Results:**

There was no statistically significant difference in demographics between the two groups. However, the Early Group had a significantly higher rate of perioperative death (9.8% vs. 1.2%, *p* = .001), RPH (48.8% vs. 14.0%, *p* < .001), and reperfusion pulmonary edema (18.3% vs. 2.9%, *p* < .001). The median follow‐up time was 66.0 months, and overall survival rates at 5, 10, 15, and 18 years after PEA were 91.2%, 83.9%, 64.5%, and 46.0%, respectively. Age and postoperative systolic pulmonary artery pressure (sPAP) were independently related to long‐term outcomes in the multivariate Cox analyses. Patients with postoperative sPAP less than 46 mm Hg had a higher chance of survival.

**Conclusions:**

PEA improved CTEPH hemodynamics immediately and had a positive effect on long‐term survival. Patients with postoperative sPAP ≥ 46 mm Hg indicate clinically significant RPH and have a lower long‐term survival rate.

## 1 INTRODUCTION

Pulmonary endarterectomy (PEA) is recommended as the gold standard treatment for chronic thromboembolic pulmonary hypertension (CTEPH),^1^, ^2^ with hemodynamic postoperative normalization for major candidates and good long‐term results.^3^, ^4^, ^5^, ^6^ However, due to the complexity of the operation and perioperative management, PEAs are primarily performed at a few high‐volume centers in the United States^7^ and Europe.^5^, ^8^

According to a meta‐analysis, despite being a curative treatment for many, a quarter of patients still present with residual pulmonary hypertension (RPH) after surgery.^9^ The increased morbidity and mortality associated with RPH should be taken seriously. Furthermore, there are no specific pulmonary hemodynamics criteria for RPH, and few data from low‐volume centers revealed that patients with immediate postoperative pulmonary vascular resistance (PVR) of 590 dynes·s/cm^5^ in an Australian report,^10^ and postoperative mean pulmonary artery pressure (mPAP) of 34 mm Hg in a Japanese study^4^ as the threshold of favorable long‐term outcome, respectively.

This study aimed to investigate the short‐ and long‐term outcomes after PEA and prognostic factors for a cohort of 253 consecutive patients with CTEPH in China. The optimal cut‐off value was analyzed for postoperative systolic PAP (sPAP), which was significant in the Cox model, in the aspect of long‐term survival.

## 2 METHODS

### 2.1 Study population

From March 1997 to December 2021, 282 patients underwent PEA at our facility in a row. We excluded 23 patients with pulmonary artery sarcoma^11^ and 6 patients with pulmonary arteritis. Finally, this study included 253 CTEPH patients. There were no more than 10 PEAs each year before 2015. According to the time and annual volume of PEA surgery, patients were categorized into two groups: the Early Group included 82 patients operated on from March 1997 to December 2014, and the Late Group included 171 patients operated on between January 2015 and December 2021. This study adhered to the World Health Organization's Declaration of Helsinki ethical standards. The Institutional Review Board of Fuwai Hospital approved the protocol (No.: 2018‐991).

### 2.2 Surgical technique

A multidisciplinary team of cardiac surgeons, pulmonologists, cardiologists, radiologists, and anesthesiologists reviewed clinical and radiological data to determine suitability for PEA surgery. The PEA procedures were performed by two experienced senior surgeons (Y. S. and S. L.), similar to those developed by the University of California San Diego (UCSD) group.^12^, ^13^ The surgery also adhered to four fundamental principles: (1) the endarterectomy should always be bilateral through a median sternotomy; (2) the operation was performed with deep hypothermic circulatory arrest (DHCA) for perfect visualization, and circulatory arrest was usually limited to 20 min at a time. The details of cardiopulmonary bypass (CPB) with DHCA for PEA had been described in detail previously^14^; (3) identification of the correct dissection plane was essential; and (4) a complete endarterectomy was crucial. Other cardiac procedures were performed during the rewarming phase.

**Figure 1:**
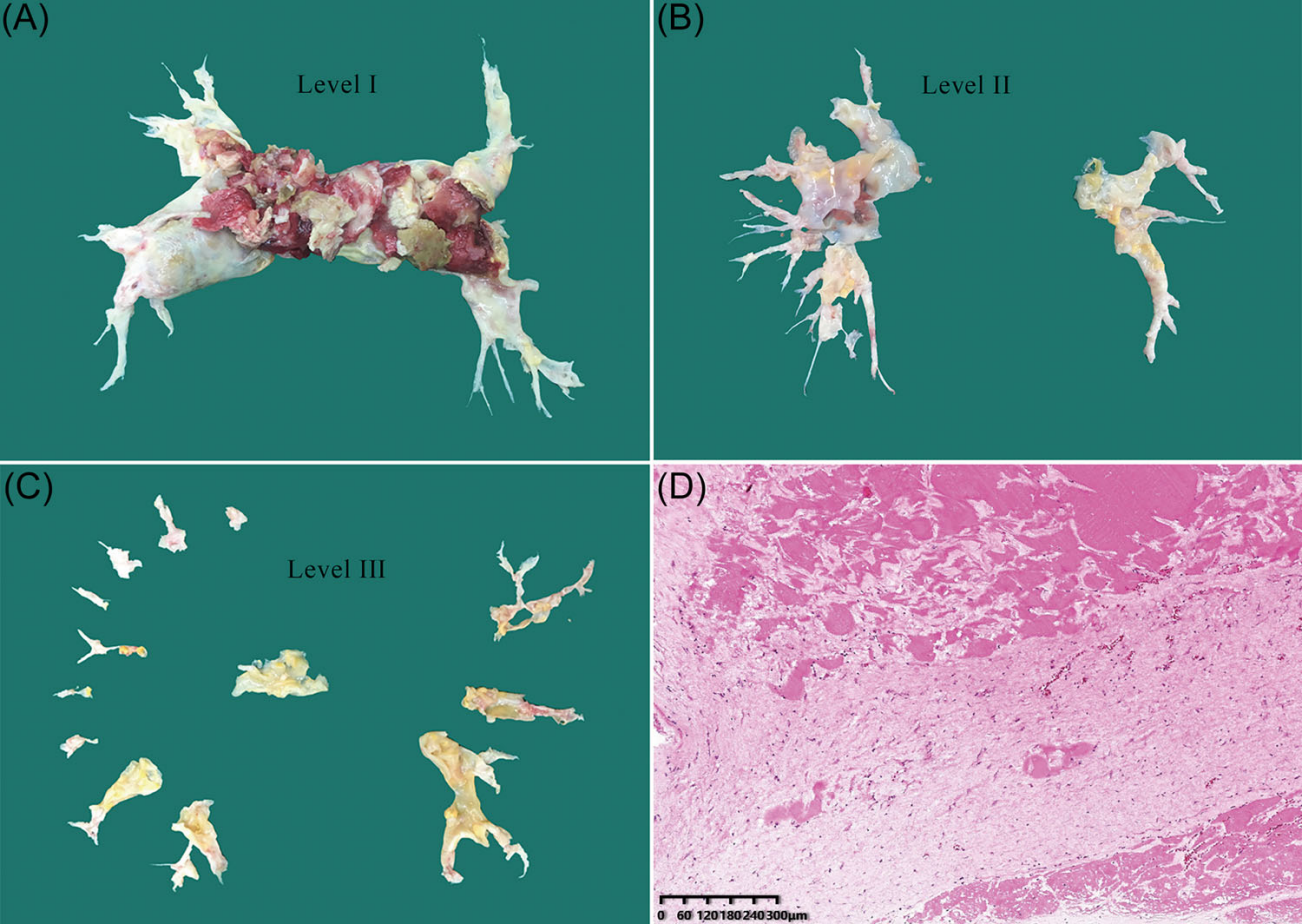
Classification of pulmonary endarterectomy disease levels and pathological image of the specimen

The endarterectomy specimens (Figure 1) were classified into four levels based on the location and morphology of the thromboembolic and vascular wall disease discovered intraoperatively.^13^ RPH after PEA was defined as mPAP greater than 25 mm Hg measured by the last Swan–Ganz catheter postoperatively.

### 2.3 Follow‐up and statistical analysis

Patients were contacted via telephone interview or office visit. Overall survival was defined as death before the end of the follow‐up period (June 30, 2022). Patients who were unable to be followed up on were censored at the time of their last visit or last known status.

Continuous variables were described as the means ± standard deviation or medians with range, according to the normality of distribution assessed using the Kolmogorov–Smirnov test. Categorical variables were expressed as frequency and percentage. Continuous variables were compared using the *t* test (independent‐samples *t* test for two groups, paired‐samples *t* test for the comparisons of change over time from the same patients), or the nonparametric test as appropriate. While Pearson *χ* ^2^ test for the comparison of categorical variables.

The impacts of clinical characteristics, hemodynamic parameters, and postoperative treatments on RPH were analyzed by logistic regression models; univariate and multivariate stepwise analyses with the Cox proportional hazard models were performed to identify the risk factors for long‐term survival (all causes). Time‐dependent receiver operator characteristic (ROC) curves at 5, 10, and 15 years were plotted for postoperative sPAP that was significant (*p* = .01) in the Cox model, using the R package “timeROC” to determine areas under the curve and the optimal cut‐off. The Kaplan–Meier method was used for survival analysis, and the log‐rank test was used to compare results. Statistical analyses were performed by using SPSS version 21.0, GraphPad Prism 7, and R 4.1.0. For all tests, *p* < .05 was considered statistically significant.

## 3 RESULTS

### 3.1 Patient characteristics

Table 1 summarizes the patient baseline characteristics in greater detail. The mean age at operation for the entire cohort was 46.5 ± 12.9 years, with 180 (71.1%) patients being male. A median of 38 months had passed since the first symptoms were observed at the time of PEA surgery. However, Early Group had significantly more male patients (80.5% vs. 66.7%, *p* = .02), longer time between symptoms and diagnosis (15.5 vs. 7.0 months, *p* < .001), shorter time from diagnosis to operation (8.0 vs. 18.0 months, *p* < .001), and more hemoptysis (39.0% vs. 24.6%, *p* = .02). There was no significant difference in combined disease between the two groups. The N‐terminal pro‐B‐type natriuretic peptide (NT pro‐BNP) was higher in the Early Group (1872.0 pg/ml) than in the Late Group (526.0 pg/ml, *p* = .001). Meanwhile, more patients in Late Group received PAH‐targeted therapy (78.9% vs. 12.2%, *p* < .001) and rivaroxaban anticoagulation (66.7% vs. 7.3%, *p* < .001).

**Table 1:** Demographics of patients

Characteristic	All (*n* = 253)	Group 1 (*n* = 82)	Group 2 (*n* = 171)	*p* Value
Age (years)	46.5 ± 12.9	44.8 ± 11.9	47.4 ± 13.3	.13
Male	180 (71.1%)	66 (80.5%)	114 (66.7%)	.02*
BMI (kg/m^2^)	23.6 ± 3.8	23.6 ± 4.0	23.6 ± 3.7	.93
Time from symptoms to diagnosis (median, months)	12.0	15.5	7.0	<.001*
Time from diagnosis to operation (median, months)	12.0	8.0	18.0	<.001*
Time from symptoms to operation (median, months)	38.0	37.0	39.0	.54
Clinical manifestation				
Short of breath	248 (98.0%)	81 (98.8%)	167 (97.7%)	.91
Thoracalgia	21 (8.3%)	8 (9.8%)	13 (7.6%)	.56
Hemoptysis	74 (29.2%)	32 (39.0%)	42 (24.6%)	.02*
Syncope	47 (18.6%)	20 (24.4%)	27 (15.8%)	.10
Combined disease				
History of acute VTE	164 (64.8%)	49 (59.8%)	115 (67.3%)	.24
Hypertension	28 (11.1%)	8 (9.8%)	20 (11.7%)	.65
CHD	22 (8.7%)	3 (3.7%)	19 (11.1%)	.05
Atrial fibrillation	16 (6.3%)	7 (8.5%)	9 (5.3%)	.32
COPD	25 (9.9%)	7 (8.5%)	18 (10.5%)	.62
Rheumatic disease	23 (9.1%)	5 (6.1%)	18 (10.5%)	.25
NT‐pro‐BNP (median, pg/ml)	643.5	1872.0	526.0	.001*
PAH‐targeted treatment	145 (57.3%)	10 (12.2%)	135 (78.9%)	<.001*
Anticoagulation				<.001*
Warfarin	111 (43.9%)	58 (70.7%)	53 (31.0%)	
Rivaroxaban	120 (47.4%)	6 (7.3%)	114 (66.7%)	
Non	22 (8.7%)	18 (22.0%)	4 (2.3%)	

Abbreviations: BMI, body mass index; CHD, coronary heart disease; COPD, chronic obstructive pulmonary disease; NT‐pro‐BNP, N‐terminal pro‐B‐type natriuretic peptide; PAH, pulmonary artery hypertension; VTE, venous thrombosis embolism.

*p < .05.

### 3.2 Surgical procedures

Table 2 shows the operational data and perioperative outcomes. In comparison to the Early Group, the incidence of UCSD level III disease increased from 15.9% to 16.4% in the Late Group, although not statistically significant. The two groups differed significantly on CPB time, with a longer CPB time in the Late Group (227.1 ± 47.6 vs. 201.6 ± 49.0 minutes, *p* < .001) because they all experienced longer cooling periods (the nasopharyngeal temperature reached 18°C) and rewarming period.

**Table 2:** Data from operations and perioperative outcomes

Characteristics	All (*n* = 253)	Group 1 (*n* = 82)	Group 2 (*n* = 171)	*p* Value
Concomitant cardiac procedures				
CABG	17 (6.7%)	3 (3.7%)	14 (8.2%)	.18
TV repair	11 (4.3%)	6 (7.3%)	5 (2.9%)	.20
Right heart thrombus resection	8 (3.2%)	1 (1.2%)	7 (4.1%)	.40
ASD	2 (0.8%)	0	2 (1.2%)	‐
UCSD classification				.92
Level I/II	212 (83.8%)	69 (84.1%)	143 (83.6%)	
Level III	41 (16.2%)	13 (15.9%)	28 (16.4%)	
CPB time (mins)	218.9 ± 49.4	201.6 ± 49.0	227.1 ± 47.6	<.001*
AC time (min)	105.7 ± 28.0	109.9 ± 30.5	103.7 ± 26.6	.10
DHCA time (min)	38.3 ± 15.4	41.2 ± 21.1	37.6 ± 13.6	.31
Postoperative MV time (median, h)	47.0	61.5	47.0	.78
Postoperative ICU stay (median, days)	6.0	6.0	5.0	.58
Postoperative hospital stay (median, days)	12.0	13.0	12.0	.61
Complications				
Perioperative death	10 (4.0%)	8 (9.8%)	2 (1.2%)	.001*
Residual pulmonary hypertension	64 (25.3%)	40 (48.8%)	24 (14.0%)	<.001*
Delirium	36 (14.2%)	16 (19.5%)	20 (11.7%)	.10
Lung infection	25 (9.9%)	11 (13.4%)	14 (8.2%)	.19
Reperfusion pulmonary edema	20 (7.9%)	15 (18.3%)	5 (2.9%)	<.001*
Pericardial effusion	17 (6.7%)	7 (8.5%)	10 (5.8%)	.42
Tracheotomy	11 (4.3%)	3 (3.7%)	8 (4.7%)	.97
Pulmonary hypertensive crisis	10 (4.0%)	8 (9.8%)	2 (1.2%)	.003*
Pulmonary hemorrhage	10 (4.0%)	4 (4.9%)	6 (3.5%)	.86
ECMO	7 (2.8%)	5 (6.1%)	2 (1.2%)	.07

Abbreviations: AC, aortic clamping; ASD, atrial septal defect; CABG, coronary artery bypass grafting; CPB, cardiopulmonary bypass; DHCA, deep hypothermic circulatory arrest; ECMO, extracorporeal membrane oxygenation; ICU, intensive care unit; MV, mechanical ventilation; SD, standard deviation; TV, tricuspid valve; UCSD, University of California San Diego.

*p < .05.

### 3.3 Early results

**Figure 2:**
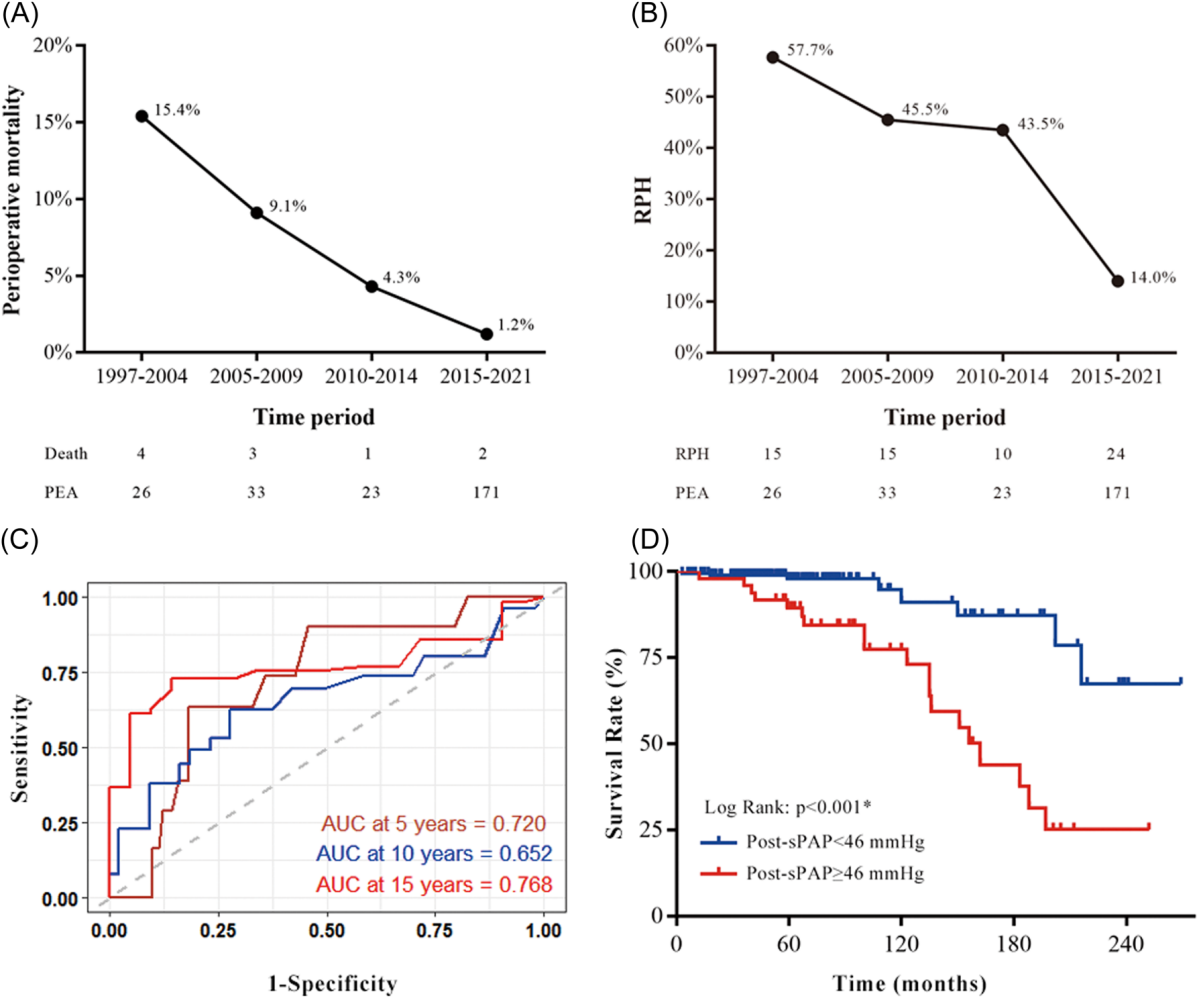
Perioperative results according to periods. (A) The perioperative mortality. (B) The RPH rates according to periods. (C) Time‐dependent ROC curves for prediction of long‐term survival performed for post‐sPAP. (D) The Kaplan–Meier survival curves for patients post‐sPAP < 46 mm Hg and post‐sPAP ≥ 46 mm Hg. AUC, are under the curve; post‐sPAP, postoperative systolic pulmonary artery pressure; ROC, receiver‐operating curve; RPH, residual pulmonary hypertension

A total of 10 (4.0%) patients died in‐hospital, with perioperative death significantly higher in the Early Group (9.8% vs. 1.2%, *p* = .001). RPH (n = 6), pulmonary bleeding (*n* = 2), sepsis (*n* = 1), and myelodysplastic syndromes (*n* = 1) were the causes of in‐hospital death. The in‐hospital mortality rate and RPH rate (Figure 2A,B) improved markedly over the years, and 171 patients were operated on between 2015 and 2021, with an in‐hospital mortality rate of 1.2% and an RPH rate of 14.0%. As shown in Table 2, the Late Group outperformed the Early Group in terms of perioperative outcomes (time in intensive care unit, RPH, reperfusion pulmonary edema, and pulmonary hypertensive crisis). On multivariate logistic regression analysis, operation before 2015 was an independent predictor for RPH after PEA (odd ratio [OR] 13.14, 95% confidence interval [CI]: 2.10–82.09, *p* = .006; see Supporting Information: Table S1).

Hemodynamic parameters at baseline, and postoperatively are listed in Supporting Information: Table S2. They were markedly improved for both groups after surgery (see Supporting Information: Figure S1). Preoperatively, there was no significant difference in pulmonary hemodynamics between the two groups, except for higher sPAP and PVR in the Late Group. Compared to the Early Group, patients in the Late Group showed significantly more reduction in sPAP (*p* < .001) and mPAP (*p* < .001) postoperatively.

### 3.4 Late survival

The median follow‐up time was 66.0 (3–269) months, and at the end of the study, 27 (11.1%) patients were dead due to right heart failure (*n* = 13), natural mortality (*n* = 6), acute pulmonary embolism (APE) (*n* = 2), bleeding (*n* = 2), stomach cancer (*n* = 1), parkinsonism (*n* = 1), and unknown (*n* = 2); meanwhile, 12 (4.9%) were lost to follow‐up. Supporting Information: Tables S2 and S3 and Figure S2 show detailed improvements in hemodynamic indicators and exercise capacity for both groups. Overall survival after PEA was 91.2%, 83.9%, 64.5%, and 46.0% at 5, 10, 15, and 18 years, respectively. Hospital survivorship rates at 5, 10, 15, and 18 years were 95.0%, 87.3%, 67.1%, and 47.9%, respectively.

For the whole cohort, univariate analyses identified several significant predictors for long‐term survival as reported in Table 3. In the multivariate analyses, age (hazard ratio [HR] 1.06, 95% CI: 1.02–1.11, *p* = .007), post‐sPAP (HR 1.08, 95% CI: 1.02–1.15, *p* = .01) and residual/recurrence PH (HR 3.75, 95% CI: 1.13–12.44, *p* = .03) were independently related to long‐term outcome. Time‐dependent ROC curves at 5, 10, and 15 years for prediction of long‐term survival were performed for post‐sPAP (Figure 2C). The area under the curve at 5, 10, and 15 years was 0.72, 0.65, and 0.77, respectively. Finally, groups were formed based on the optimal ROC cut‐off value (46 mm Hg), and the Kaplan–Meier survival curves for the two cohorts are shown in Figure 2D. Patients with sPAP < 46 mm Hg after surgery had a survival benefit, with conditional 5‐, 10‐, and 15‐year survival rates of 97.9%, 91.1%, and 87.3% compared to 89.4%, 77.4%, and 37.6% (*p* < .001).

**Table 3:** Survival analysis for CTEPH (whole cohort)

Covariate	Univariate analysis		Multivariate analysis	
	HR (95% CI^a^)	*p* Value	HR (95% CI^a^)	*p* Value
Age	1.05 (1.01–1.10)	.01*	1.06 (1.02–1.11)	.007*
Sex	0.52 (0.22–1.22)	.14		
BMI	0.92 (0.81–1.06)	.26		
Duration from attack to operation	1.00 (0.99–1.01)	.59		
Pre‐NYHA	1.11 (0.37–3.34)	.85		
Pre‐PAH‐targeted treatment	1.23 (0.39–3.88)	.72		
Pre‐NT pro‐BNP	1.00 (0.99–1.00)	.46		
Pre‐sPAP	1.02 (1.00–1.04)	.04*	1.02 (1.00–1.04)	.13
Pre‐mPAP	1.03 (0.99–1.07)	.09		
Pre‐CI^b^	1.12 (0.42–2.94)	.83		
Pre‐PVR	1.00 (0.99–1.00)	.64		
UCSD type	1.04 (0.36–3.04)	.94		
Circulatory arrest time	1.01 (0.99–1.04)	.37		
Post‐sPAP	1.05 (1.03–1.07)	<.001*	1.08 (1.02–1.15)	.01*
Post‐dPAP	1.09 (1.04–1.15)	.001*	0.96 (0.85–1.08)	.49
Post‐mPAP	1.06 (1.03–1.09)	<.001*	0.93 (0.79–1.09)	.35
Post‐CI^b^	0.65 (0.08–5.49)	.70		
Post‐PVR	1.00 (0.99–1.01)	.84		
Residual/recurrence PH	4.43 (1.86–10.52)	.001*	3.75 (1.13–12.44)	.03
Post PAH‐targeted treatment	2.07 (0.61–7.00)	.24		

Abbreviations: BMI, body mass index; CIa, confidence interval; CIb, cardiac index; dPAP, diastolic pulmonary artery pressure; HR, hazard ratio; mPAP, mean pulmonary artery pressure; NT pro‐BNP, N‐terminal pro‐B‐type natriuretic peptide; NYHA, New York Heart Association; PAH, pulmonary artery hypertension; PH, pulmonary hypertension; Pre, preoperative; Post, postoperative; PVR, pulmonary vascular resistance; sPAP, systolic pulmonary artery pressure; TAPSE, tricuspid annular plane systolic excursion; UCSD, University of California San Diego; VTE, venous thrombosis embolism.

*p < .05.

## 4 DISCUSSION

To the best of our knowledge, this is the largest single‐center retrospective cohort study of 253 CTEPH patients undergoing PEA in Asia. It shows that the short‐ and long‐term outcomes are excellent, with lower perioperative mortality and lower RPH rates in the Late group compared to the Early Group, indicating a learning curve in our center. Furthermore, analyses confirmed that postoperative sPAP ≥ 46 mm Hg was negatively associated with long‐term survival.

The basic clinical status of CTEPH patients in our center differs from previous studies in some ways. Patients in our hospital (46.5 ± 12.9 years old) were slightly younger, while 51.6 years old in the UCSD group,^7^ 57 ± 15 years old in the United Kingdom national cohort,^6^ and 56 years old in the Japan group.^15^ In this study, men made up 71.1% of the population. However, the ratio of male/female was almost equal in high‐volume centers.^6^, ^7^, ^8^ The age and gender differences between countries may be due to patients' surgery intentions, and a large number of young men, who represented the majority of productivity in developing countries, received surgery in the early years. A high proportion of young rheumatic patients combined with CTEPH (9.1%) might lower the average age. Although advanced age is not a contraindication to surgery in an experienced center,^16^ junior patients may be more preferred to receive surgery in low‐volume centers. Jensen et al.^17^ suggested that the use of PAH‐targeted drugs was associated with a delay in the time to referral for PEA surgery with no significant differences in postoperative outcomes or hemodynamics. Nonetheless, CTEPH patients were more likely to receive PAH medical therapies in China, which could lengthen the time between diagnosis and operation. Consistent with western countries in this cohort, there was an increase in the use of direct oral anticoagulants (DOACs) during the study period, even though there is little data on the safety and efficacy of DOACs for inoperable CTEPH patients.^18^

The surgical result in our center had witnessed an obvious learning curve (Figure 2); these experience‐related operation outcomes have also been reported in Europe and the United States.^3^, ^6^, ^7^, ^19^ Gratifyingly, the perioperative mortality rate in the Late Group was 1.2%, with a 14.0% RPH rate, which is comparable to other expert high‐volume centers around the world.^6^, ^7^ With improvements in surgical techniques, perioperative mortality has fallen from almost 20% in the early years to <2% at UCSD,^20^ the biggest CTEPH center in the world. Although a curative operation for many, 25% of patients experience RPH after PEA according to a meta‐analysis.^9^ The dramatic improvement in morbidity and mortality of the Late Group could be attributed to the following aspects: First, as international experts pointed out, one of the most important factors contributing to excellent results is case volume.^5^, ^21^ From 2015 onwards, there were 30 to 35 PEA procedures each year, with no more than 10 cases in the Early Group. All PEA procedures were carried out by two experienced senior surgeons, which shortened the learning curve. The more experience and training a surgeon had, the easier it was to identify the correct plane of dissection, the short operation time, and the less pulmonary vascular injury. Second, the bloodless visualization with DHCA^14^ and the use of specialized surgical instruments help to extract more distal scars. A complete endarterectomy is crucial for satisfying perioperative and long‐term results. Third, we discovered that venting the heart during the fibrillation period, maintaining patients in a negative fluid balance for the first 24 h, limiting the postoperative cardiac index to around 2.0 L/min/m^2^, and using effective positive pressure ventilation reduced the rate of reperfusion pulmonary edema. Finally, the lack of a licensed targeted therapy (riociguat) for the treatment of CTEPH may have contributed to poorer perioperative outcomes in the Early Group of our study.

CTEPH is a fatal complication that affects about 4% of patients after APE. It is crucial to diagnose CTEPH without any delay in patients with history of APE, due to proper diagnosis enables PEA and/or riociguat that improves their prognosis.^22^ As is known to all, surgery is the gold standard treatment for CTEPH patients. There are few reports due to the scarcity of specialized centers with experience in PEA. The current 5‐ and 10‐year survival rates in this study are comparable to the survival rates reported from other experienced centers in America, Europe, and Japan.^3^, ^7^, ^15^, ^23^ The latest study from Germany, including 499 consecutive patients who underwent PEA over 20 years, has reported overall survival and survival after discharge at 15 years were 59.2% and 65.6%, respectively.^3^ Our overall survival of 64.5% and hospital survivors of 67.1% at 15 years were both higher. The differences could be attributed to our cohorts' younger age (46.5 ± 12.9 vs. 57.5 ± 14.0 years), lower perioperative death (4.0% vs. 8.4%), and lower RPH rate (25.3% vs. 34.1%) in our cohort.

The level of pulmonary hemodynamic values could be a predictor of hospital mortality and long‐term outcome. Although the thresholds were discrepant in many previous publications, they all indicated that PVR was a very good predictor of perioperative mortality.^4^, ^7^, ^24^, ^25^ However, only a few data from low‐volume centers revealed predictive factors of long‐term survival, Skoro‐Sajer et al.^10^ revealed that patients with immediate postoperative PVR < 590 dynes·s/cm^5^ had a better long‐term outcome (*p* < .0001) in 110 PEA patients. A Japanese study including 77 cases recommended a postoperative mPAP of 34 mm Hg as the cut‐off value for late adverse events.^4^ Our study, which included 253 PEA cases and a longer follow‐up period than the above two, confirmed that age and sPAP after surgery were associated with late death. According to time‐dependent ROC curves, a Post‐sPAP of 46 mm Hg is the cut‐off value for long‐term survival. Because there is no agreement on the definition of RPH after PEA surgery, we, therefore, propose that Post‐sPAP ≥46 mm Hg represents clinically significant RPH. According to the 2018 Cologne Consensus Conference,^1^ these patients should begin treatment with riociguat, the first licensed therapy for the treatment of residual/recurrent pulmonary hypertension after PEA,^26^ and/or receive balloon pulmonary angioplasty as soon as possible.

An inherent shortcoming of this study is its retrospectively observational single‐center design. However, this cohort represents the largest sample size for a single institution for 25 years in Asia, and more than 95% of patients had finished the follow‐up. Another limitation of our study is the lack of complete data for some patients who underwent surgery in the 20th century and those discharged back to their local hospitals.

## 5 CONCLUSIONS

In conclusion, PEA improved hemodynamics immediately and had a positive effect on long‐term survival in CTEPH patients. With the accumulation of therapy experiences, better hemodynamic improvements and perioperative mortality results will be achieved in low‐ or medium‐volume centers. Patients with postoperative sPAP ≥ 46 mm Hg indicate clinically significant RPH and poor long‐term survival rates and should be treated with active medical or interventional therapy.

## AUTHOR CONTRIBUTIONS

Wu Song analyzed most of the data and prepared the manuscript. Jiade Zhu and ZhaoJi Zhong collected the data and carried out additional analyses. Yunhu Song and Sheng Liu performed operations for patients and designed and coordinated the study. All authors read and approved the final manuscript.

## CONFLICT OF INTEREST

The authors declare no conflict of interest.

## Supporting information

Supporting information.

## Data Availability

The data sets generated and/or analyzed during the current study are not publicly available but are available from the corresponding author on reasonable request.
