# Supplementary material for: Long‐term outcome prediction for chronic thromboembolic pulmonary hypertension after pulmonary endarterectomy
Source: Clin Cardiol. 2022 Sep 7;45(12):1255–63. doi: 10.1002/clc.23900 (PMC9748755; doi:10.1002/clc.23900)

**Supplementary**-**Table S1**. Logistic regression residual pulmonary hypertension analysis for CTEPH (whole cohort)

| Covariate | Univariate analysis | | Multivariate analysis | |
| --- | --- | --- | --- | --- |
|  | OR (95% CI^a^) | p value | OR (95% CI^a^) | p value |
| Age | 1.02 (0.99-1.04) | 0.16 |  |  |
| Sex | 1.56 (0.85-2.85) | 0.15 |  |  |
| BMI | 0.90 (0.82-1.00) | **0.04*** | 0.97 (0.85-1.10) | 0.60 |
| Duration from attack to operation | 1.00 (0.99-1.00) | 0.77 |  |  |
| VTE history | 0.86 (0.45-1.64) | 0.65 |  |  |
| Anticoagulant (Rivaroxaban) | 0.42 (0.21-0.85) | **0.02*** | 0.77 (0.28-2.09) | 0.60 |
| Pre PAH-targeted treatment | 0.37 (0.21-0.66) | **0.001*** | 1.05 (0.29-3.85) | 0.94 |
| Pre-sPAP | 1.02 (1.00-1.03) | **0.04*** | 0.94 (0.88-1.02) | 0.13 |
| Pre-dPAP | 1.05 (1.01-1.08) | **0.006*** | 0.86 (0.73-1.01) | 0.07 |
| Pre-mPAP | 1.03 (1.01-1.06) | **0.01*** | 1.21 (0.98-1.50) | 0.08 |
| Pre-CO | 0.85 (0.66-1.11) | 0.23 |  |  |
| Pre-CI^b^ | 0.74 (0.44-1.23) | 0.25 |  |  |
| Pre-PVR | 1.001 (1.001-1.002) | **0.002*** | 1.001 (0.999-1.003) | 0.18 |
| Operation before 2015 | 5.83 (3.17-10.75) | **＜0.001*** | 13.14 (2.10-82.09) | **0.006*** |
| UCSD type III | 1.27 (0.61-2.67) | 0.52 |  |  |
| Circulatory arrest time | 1.02 (1.00-1.04) | 0.05 | 1.01 (0.98-1.04) | 0.64 |

BMI: body mass index; CI^a^: confidence interval; CI^b^: cardiac index; CO: cardiac output; dPAP: diastolic pulmonary artery pressure; mPAP: mean pulmonary artery pressure; OR: odds ratio; PAH: pulmonary artery hypertension; Pre: preoperative; PVR: pulmonary vascular resistance; sPAP: systolic pulmonary artery pressure; TAPSE: tricuspid annular plane systolic excursion; UCSD: University of California, San Diego; VTE: venous thrombosis embolism.

**Supplementary**-**Table S2**. Hemodynamics in different periods

| **Variable** | **All (n = 253)** | **Group 1 (n = 82)** | **Group 2 (n = 171)** | **p value** |
| --- | --- | --- | --- | --- |
| sPAP (mmHg) |  |  |  |  |
| Preoperative | 83.0 ± 21.4 | 87.5 ± 18.3 | 80.9 ± 22.5 | **0.02*** |
| Postoperative | 40.0 ± 16.5 | 51.7 ± 20.0 | 34.2 ± 10.6 | **<0.001*** |
| Follow-up (Eco) | 40.7 ± 17.7 | 51.3 ± 23.7 (n = 36) | 37.3 ± 13.9 (n = 115) | **<0.001*** |
| mPAP (mmHg) |  |  |  |  |
| Preoperative | 47.8 ± 12.1 | 50.0 ± 10.9 | 46.8 ± 12.6 | 0.05 |
| Postoperative | 24.0 ± 9.7 | 30.0 ± 12.5 | 21.1 ± 6.2 | **<0.001*** |
| CO (L/min) |  |  |  |  |
| Preoperative | 4.7 ± 1.4 | 4.7 ± 1.5 | 4.7 ± 1.3 | 0.87 |
| Postoperative | 4.3 ± 1.1 | 6.6 ± 1.8 | 4.2 ± 1.0 | **<0.001*** |
| CI (L/min/m^2^) |  |  |  |  |
| Preoperative | 2.6 ± 0.7 | 2.4 ± 0.8 | 2.6 ± 0.6 | 0.16 |
| Postoperative | 2.3 ± 0.6 | 3.5 ± 0.9 | 2.3 ± 0.5 | **<0.001*** |
| PVR (median, dyn.s.cm^−5^) |  |  |  |  |
| Preoperative | 721.6 | 788.2 | 668.4 | **0.04*** |
| Postoperative | 212.0 | 245.6 | 211.5 | 0.70 |

CI: cardiac index; CO: cardiac output; Ech: echocardiography; mPAP: mean pulmonary artery pressure; PVR: pulmonary vascular resistance; sPAP: systolic pulmonary artery pressure.

**Supplementary**-**Table S3**. Cardiac function in different periods

| **Variable** | **All (n=253)** | **Group 1 (n=82)** | **Group 2 (n=171)** | **p value** |
| --- | --- | --- | --- | --- |
| Preoperative Functional class NYHA (n, %) |  |  |  | **＜0.001*** |
| I/II | 69 (27.3%) | 9 (11.0%) | 60 (35.1%) |  |
| III/IV | 184 (72.7%) | 73 (89.0%) | 111 (64.9%) |  |
| Last follow-up Functional class NYHA (n, %) |  |  |  | **0.04*** |
| I/II | 203 (93.5%) | 44 (86.3%) | 159 (95.8%) |  |
| III/IV | 14 (6.5%) | 7 (13.7%) | 7 (4.2%) |  |
| 6MWT (m) |  |  |  |  |
| Preoperative | 392.5±95.1 (n=84) | 389.4±97.0 | 426.4±66.6 | 0.33 |
| Follow-up | 455.3±72.7 (n=86) | 454.3±72.2 | 467.3±83.4 | 0.65 |
| RVD (mm) |  |  |  |  |
| Preoperative | 33.9±8.1 | 35.6±8.0 | 33.2±8.1 | **0.03*** |
| Postoperative | 24.6±5.1 | 25.3±5.7 | 24.3±4.8 | 0.21 |
| Follow-up | 24.1±5.1 | 25.0±6.5 | 23.9±4.6 | 0.32 |

NYHA: New York Heart Association; 6MWT: six-minute walk test; RVD: right ventricular diameter.

**Supplementary-Figure S1.** The preoperative and postoperative haemodynamics for two groups. CI: cardiac index; CO: cardiac output; dPAP: diastolic pulmonary artery pressure; mPAP: mean pulmonary artery pressure; PVR: pulmonary vascular resistance; sPAP: systolic pulmonary artery pressure. Group 1: patients between 1997 and 2014, Group2: patients between 2015 and 2021. ** p<0.001


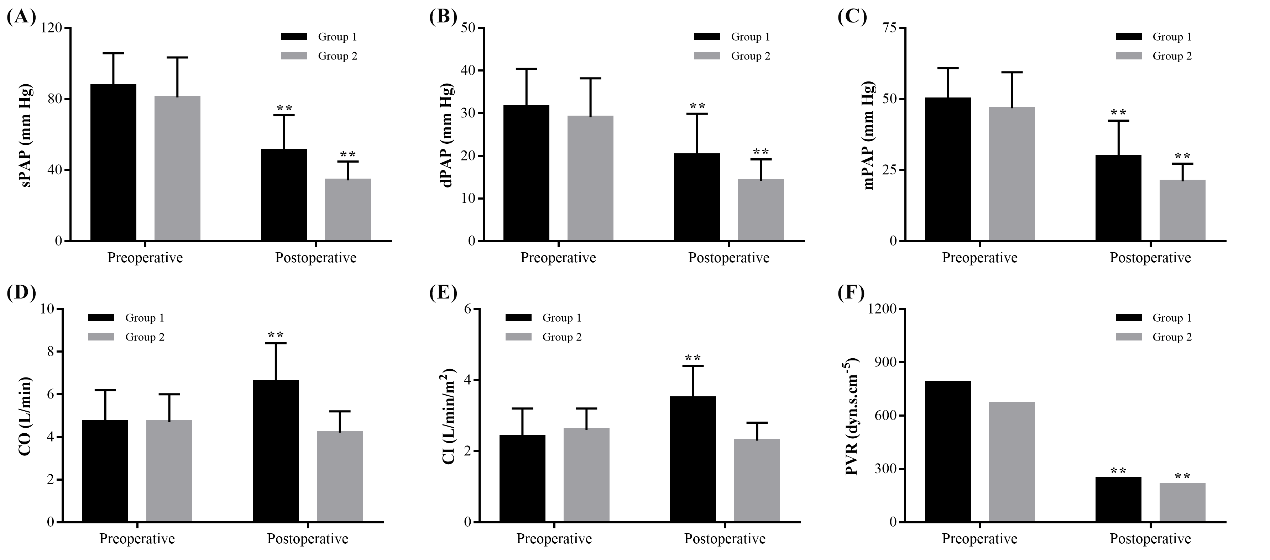


**Supplementary-Figure S2.** The preoperative and follow-up cardiac function and haemodynamics for two groups. 6MW: six-minute walk test; Eco: echocardiography; NYHA: New York Heart Association; RVD: right ventricular diameter; sPAP: systolic pulmonary artery pressure. Group 1: patients between 1997 and 2014, Group2: patients between 2015 and 2021. * p<0.05, ** p<0.001.


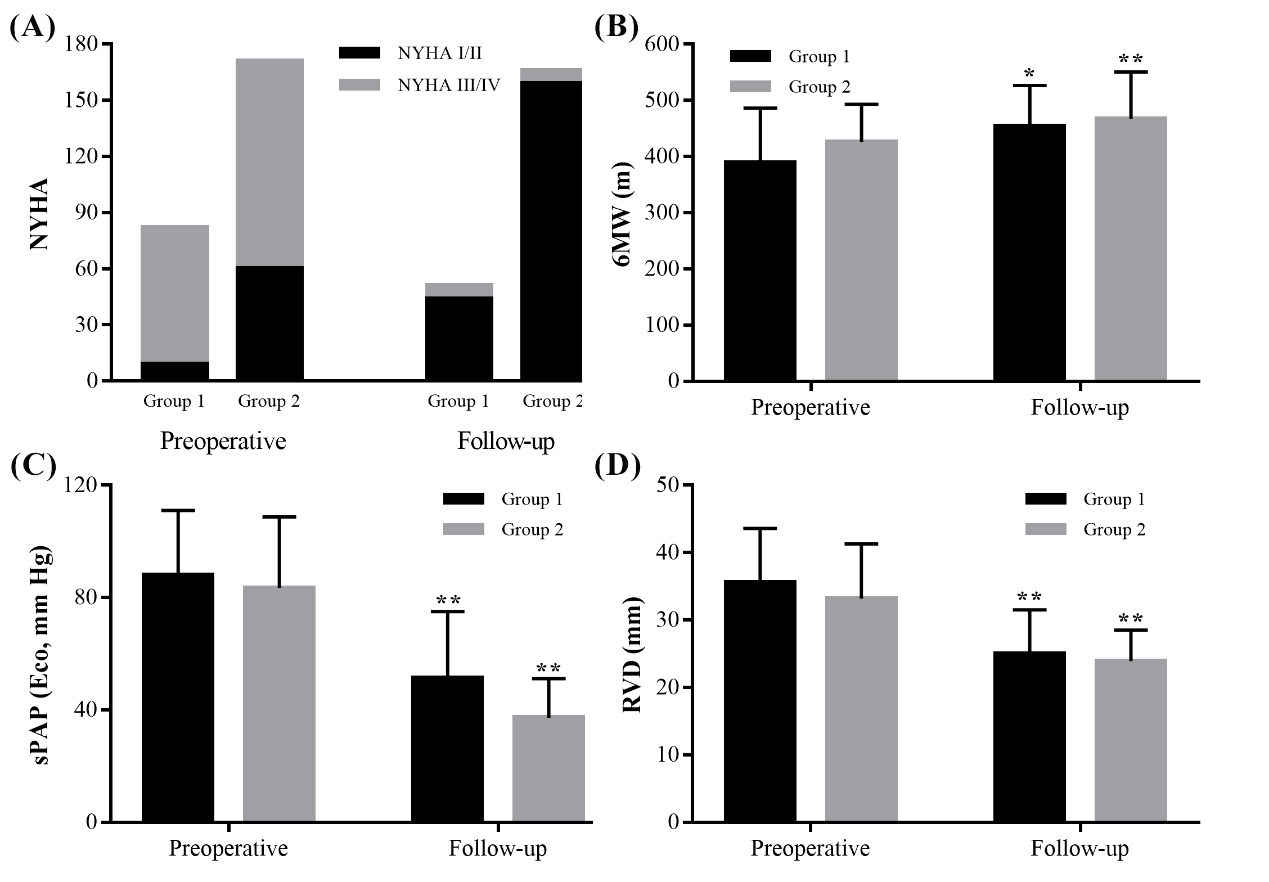

Supplement: Supplementary file 1 — Supporting information. [file CLC-45-1255-s001.docx]
